# Mechanically Robust and Highly Conductive Cellulose Nanopaper Electrodes Enabled by Polyaniline-Graphene Hybrid Networks for Flexible Supercapacitors

**DOI:** 10.1021/acsomega.6c10164

**Published:** 2026-10-09

**Authors:** Mengge Gao, Kun Liu, Ningning Tan, Yuanye Jiang, Jiansong Chen, Yaoqi Yin, Aijun Xie, Xuejun Pan, Haishun Du, Sundaram Gunasekaran

**Affiliations:** † Department of Biological Systems Engineering, 5228 University of Wisconsin-Madison, Madison, Wisconsin 53706, United States; ‡ Department of Forestry, 3078 Michigan State University, East Lansing, Michigan 48824, United States

## Abstract

Flexible, freestanding electrodes with high mechanical robustness, electrical conductivity, and electrochemical performance are highly desirable for wearable energy storage devices. Herein, a hierarchical polyaniline/partially oxidized graphene/cellulose nanopaper (PANI/po-Gr/CNP) electrode was developed by integrating redox-active PANI, conductive po-Gr, and mechanically robust cellulose nanofibers (CNFs). PANI-coated CNFs were first prepared via in situ polymerization, followed by incorporation of po-Gr nanosheets. We vacuum-filtered and dried the resulting hybrid suspension to fabricate a freestanding PANI/po-Gr/CNP electrode. Compared with the po-Gr-free PANI/CNP electrode, the optimized PANI/po-Gr/CNP electrode increased electrical conductivity from 0.16 to 1.85 S·cm^–1^ while maintaining a comparable tensile strength (∼42 MPa). In a three-electrode configuration, the optimized electrode delivered an areal capacitance of 2.74 F·cm^–2^ at 5 mV·s^–1^, corresponding to volumetric and gravimetric capacitances of 342.5 F cm^–3^ and 572.7 F·g^–1^ (based on the combined mass of PANI and po-Gr), respectively. The assembled symmetric supercapacitor delivered an areal energy density of 58.08 μWh·cm^–2^ and a volumetric energy density of 7.26 mWh·cm^–3^, while retaining 81.0% of its initial capacitance after 10,000 charge–discharge cycles. These results show that combining CNFs, PANI, and po-Gr enables a freestanding electrode with a favorable balance of mechanical robustness, electrical conductivity, and electrochemical performance, offering a promising platform for flexible, wearable supercapacitors.

## Graphical abstract

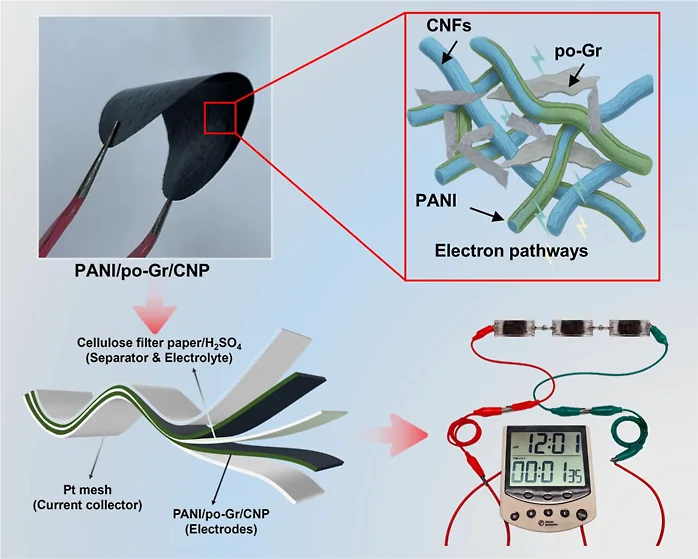

## 1 Introduction

The rapid development of wearable and portable electronics has accelerated the demand for lightweight, flexible, and mechanically robust energy storage devices.^1^ Supercapacitors are promising candidates for these applications due to their high power density, fast charge–discharge capability, and long cycling stability.^2,3^ The electrochemical performance of supercapacitors is largely determined by the electrode architecture and active materials employed. Carbon-based materials provide excellent rate capability through electric double-layer capacitance, whereas conducting polymers and transition-metal oxides deliver higher pseudo capacitance via reversible surface redox reactions.^4–9^ However, most conventional supercapacitor electrodes are fabricated on rigid metal foils, resulting in brittleness, poor flexibility, and interfacial delamination under repeated mechanical deformation.^10–12^ These limitations hinder their integration into flexible and wearable devices.

Among various pseudocapacitive materials, polyaniline (PANI) has attracted considerable attention due to its high theoretical capacitance (∼2000 F·g^–1^), low cost, facile synthesis, and tunable redox properties.^13,14^ Nevertheless, the practical application of PANI is restricted by its poor cycling stability. During repeated charge–discharge processes, the large volume expansion and contraction associated with proton doping and dedoping often induce structural cracking, loss of electrical contact, and capacitance decay.^15^ For example, pure PANI electrodes typically retain less than 40–50% of their initial capacitance after only 1000–2000 cycles.^16,17^ Incorporating carbon nanomaterials has proven effective in mitigating these issues. Tian et al. reported hierarchical PANI/N-CNT fibers showing a specific capacitance of 557 F·g^–1^ and 93% capacitance retention after 5000 cycles.^18^ Hu et al. demonstrated enhanced cycling stability (∼85% retention) using rGO-wrapped PANI nanofibers,^17^ while Xu et al. developed flexible CM-PANI/CNT composite films retaining ∼89% of initial capacitance after 5000 cycles.^19^ These studies suggest that integrating PANI with conductive and mechanically resilient nanostructures is an effective strategy for enhancing both electrochemical performance and cycling durability.

Graphene is particularly attractive for constructing conductive frameworks in flexible supercapacitor electrodes due to its high conductivity, large surface area, and exceptional mechanical strength.^20–22^ Electrochemical exfoliation provides a scalable and environmentally benign route for producing high-quality graphene with tunable surface chemistry and minimal structural damage, making it suitable for flexible supercapacitor electrodes.^23^ However, graphene nanosheets tend to restack because of strong π–π interactions and van der Waals attractions, resulting in reduced accessible surface area, hindered ion diffusion, and diminished electrochemical performance.^24^ In addition, densely packed graphene films often possess insufficient structural integrity and flexibility for long-term operation under repeated mechanical deformation.

Cellulose nanofibers (CNFs), derived from abundant and renewable biomass resources, have recently emerged as a promising building block for flexible electronics.^25–28^ Their high aspect ratio and ability to form extensive hydrogen bonds enable an entangled network that can bend while maintaining the integrity needed for a freestanding nanopaper.^29^ Recent studies have further demonstrated the versatility of engineered cellulose fibers and reassembled fiber networks in flexible functional materials.^30–32^ In hybrid electrodes, CNFs also help disperse graphene and conducting polymers and strengthen their interactions within the network.^33,34^ For example, incorporating bacterial cellulose nanofibers increased the tensile strength of MXene films from 19.3 to 88.9 MPa,^35^ while CNF-reinforced conductive hydrogels exhibited remarkable improvements in stretchability, toughness, and structural stability.^36^ These findings underscore the potential of CNFs as sustainable reinforcing scaffolds for constructing mechanically robust freestanding electrodes.

Despite recent progress, achieving high capacitance, electrical conductivity, and mechanical robustness in a single freestanding electrode remains challenging. Here, we develop a PANI/po-Gr/CNP electrode that combines the mechanical support of CNFs, the redox activity of PANI, and the conductive pathways provided by po-Gr. As illustrated in Scheme 1, PANI is polymerized on CNFs, followed by the incorporation of po-Gr nanosheets and vacuum filtration to form the freestanding electrode. The optimized electrode achieves a tensile strength of 42.5 MPa, an electrical conductivity of 1.85 S·cm^–1^, and an areal capacitance of 2.74 F·cm^–2^ at 5 mV·s^–1^. A symmetric supercapacitor assembled from these electrodes retains 81.0% of its initial capacitance after 10,000 cycles.

**Scheme 1:**
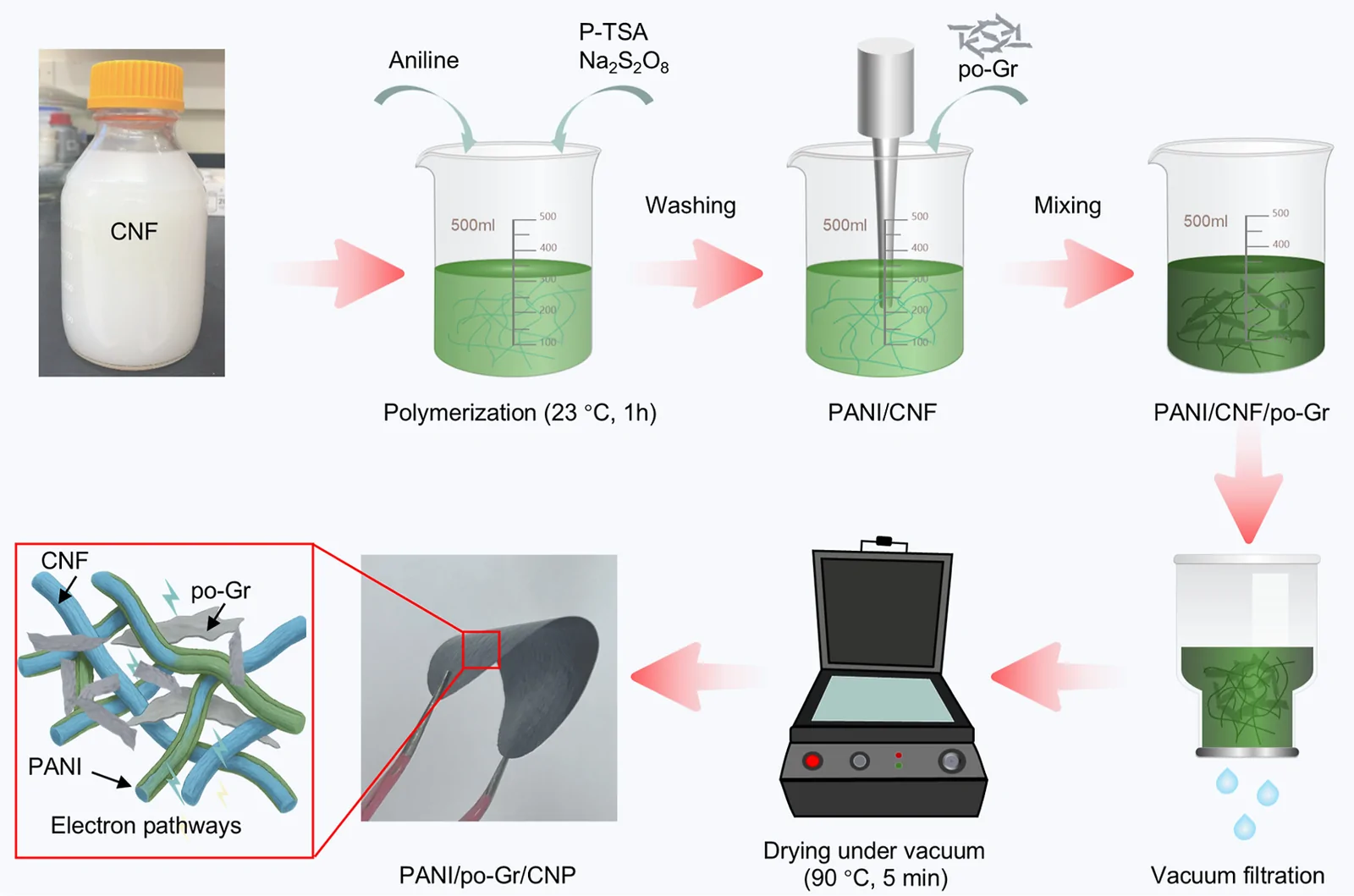
Schematic Diagram of the Preparation Process of PANI/Po-Gr/CNP

## 2 Experimental Section

### 2.1 Materials and Reagents

Cellulose nanofibers (CNFs, ∼3 wt %, Lot U31) were obtained from the University of Maine (USA). Aniline monomer (99%) was purchased from Alfa Aesar. Sodium persulfate (Na_2_S_2_O_8_), *p*-toluenesulfonic acid (*p*-TSA), and sulfuric acid (H_2_SO_4_, 95–98%) were obtained from Santa Cruz Biotechnology, Millipore Sigma, and VWR, respectively. Graphite oxide sheets were purchased from Graphite Store (USA). All chemicals were used as received without further purification.

### 2.2 Fabrication of PANI/Po-Gr/CNP Composite Films

PANI was polymerized in situ on CNFs via an oxidative chemical polymerization method A 3 wt % CNF suspension (10 g) was dispersed in 1.0 M *p*-TSA under stirring, followed by the addition of aniline monomer and mixing for 10 min. Polymerization was initiated by adding Na_2_S_2_O_8_ and allowed to proceed at room temperature for 1 h. The resulting green PANI/CNF gel was collected by vacuum filtration, thoroughly washed with DI water, redispersed to a fixed volume, and sonicated to form a homogeneous PANI/CNF suspension. To tune PANI loading, the aniline amount was adjusted from 0.2 to 0.8 mL while maintaining proportional oxidant content, yielding PANI/CNF mixtures containing 15.7–60.8 wt % PANI (Table 1).

**Table 1:** Reactant Composition in the Preparation of PANI/CNF and the Weight Ratio of PANI in the PANI/CNP

sample	CNFs (g)	aniline (mL)	Na_2_S_2_O_8_ (g)	weight ratio of PANI (%)
Pure CNP	0.3	0	0	0
PANI/CNP-1	0.3	0.2	0.24	15.7 ± 0.8
PANI/CNP-2	0.3	0.4	0.48	39.3 ± 1.9
PANI/CNP-3	0.3	0.6	0.72	50.7 ± 2.4
PANI/CNP-4	0.3	0.8	0.96	60.8 ± 3.1

Freestanding PANI/CNP samples were fabricated by vacuum-filtering the PANI/CNF suspension through a polypropylene membrane (0.45 μm pore size). The resulting wet gel was laminated between membrane sheets and dried using a plate vacuum dryer at 90 °C for 5 min, producing dense, uniform PANI/CNP films.

Po-Gr was synthesized by electrochemical exfoliation using graphite sheets as both electrodes in 0.1 M PBS (pH 7.0) under a 10 V DC potential for 30 min. The exfoliated flakes were collected via filtration (0.2 μm), washed thoroughly, redispersed by ultrasonication (500 W, 10 min), and centrifuged to obtain a stable 1 mg·mL^–1^ po-Gr dispersion. As illustrated in Scheme 1, po-Gr dispersions were incorporated into the optimized PANI/CNF suspension at loadings of 10–25 wt % based on the dry weight of the PANI/CNF matrix. The resulting mixtures were homogenized by sonication and subsequently subjected to the same filtration–drying process described above to fabricate freestanding PANI/po-Gr/CNP composite films. The incorporation of po-Gr was expected to enhance both the electrical conductivity and mechanical performance of the resulting films.

### 2.3 Materials Characterization

#### 2.3.1 Surface Chemistry

Fourier transform infrared (FT-IR) spectra were collected using a PerkinElmer Spectrum 100 spectrometer within 4000–400 cm^–1^ at a resolution of 4 cm^–1^. The surface chemical composition of the samples was characterized by X-ray photoelectron spectroscopy (XPS) using a Thermo Scientific K-Alpha spectrometer equipped with an Al Kα X-ray source.

#### 2.3.2 Morphological Characterization

CNF and PANI/CNP morphologies were observed using transmission electron microscopy (TEM, JEM-2100, 200 kV) STEM-HAADF elemental mapping was performed on a Hitachi S-5500 microscope. Surface and cross-sectional morphologies of CNP, PANI/CNP, and PANI/po-Gr/CNP were examined using a field-emission scanning electron microscope (FESEM, Apreo FE) at 2 kV. Samples were sputter-coated with gold (Leica EM ACE600) before imaging.

#### 2.3.3 Electrical Conductivity

Sheet resistance was measured using the van der Pauw four-point method with a Keithley DAQ6510 multimeter. Square specimens (1.5 × 1.5 cm^2^) were prepared, and their corners were coated with silver paint for good contact. Sheet resistance (R_s_) was calculated as

1 $$R_{s}=\frac{\pi R}{\ln2}\approx 4.53R$$

Electrical conductivity (σ) was calculated by

2 $$\sigma =\frac{1}{R_{s}\times t}$$

where *t* is film thickness. Three replicates were tested per sample.

#### 2.3.4 Electrochemical Characterization

Electrochemical measurements were carried out on a CHI 660E workstation using 1.0 M H_2_SO_4_ as the electrolyte. In the three-electrode configuration, the PANI/CNP or PANI/po-Gr/CNP (1 × 1 cm^2^) was directly used as the working electrode, while a platinum mesh and an Ag/AgCl (3 M KCl) electrode served as the counter and reference electrodes, respectively. Cyclic voltammetry (CV) was performed within a potential window of 0–0.8 V at scan rates ranging from 5 to 100 mV·s^–1^. Galvanostatic charge–discharge (GCD) tests were conducted over the same potential range with current densities between 5 and 100 mA·cm^–2^. Electrochemical impedance spectroscopy (EIS) was recorded over a frequency range of 100 kHz to 0.01 Hz with an amplitude of 5 mV.

Specific capacitance (C_sp_) was calculated using

3 $$C_{sp}=\frac{\int IdV}{\nu A\Delta V}$$

Symmetric supercapacitors were assembled using two identical PANI/po-Gr/CNP electrodes (1 × 1 cm^2^, 80 μm thickness, 7.9 mg). A cellulose separator soaked in 1.0 M H_2_SO_4_ was used. Electrochemical testing followed the same protocol as above.

Capacitance (C_cell_)­

4 $$C_{cell}=\frac{\int IdV}{\nu A\Delta V}$$

Energy and power densities

5 $$E=\frac{C_{cell}{\left(\Delta V\right)}^{2}}{2\times 3600}$$

6 $$P=\frac{3.6E\nu }{\Delta V}$$

#### 2.3.5 Mechanical Testing

Mechanical properties were evaluated using a universal testing machine (F305, Mark-10). Rectangular specimens (50 × 5 mm) were pulled at 5 mm·min^–1^. Tensile strength and elongation at break were recorded, and five replicates were tested for each sample.

## 3 Results and Discussion

### 3.1 Mechanical and Electrical Properties

The mechanical and electrical properties of the composite films were systematically investigated to identify an optimal electrode composition. As shown in Figure 1a, pristine CNP exhibited the highest tensile strength (112.8 MPa) and a fracture strain of approximately 14%, owing to the highly entangled CNF network and extensive hydrogen-bonding interactions. Incorporation of PANI progressively reduced both tensile strength and elongation at break. The disruption of interfibrillar hydrogen bonding and the introduction of relatively rigid PANI domains weakened the cellulose network, resulting in reduced tensile strengths. Nevertheless, the incorporation of PANI significantly improved the electrical conductivity of the films, increasing from 0.01 S·cm^–1^ for PANI/CNP-1 to 0.45 S·cm^–1^ for PANI/CNP-4 (Figure 1b). This increase can be attributed to the formation of increasingly interconnected conductive pathways within the CNF network. Considering the balance between mechanical robustness and electrical conductivity, PANI/CNP-3, containing 50.7 wt % PANI, was selected as the optimal composition for subsequent po-Gr incorporation. This composition maintained a tensile strength of 41.9 MPa while exhibiting a conductivity of 0.16 S·cm^–1^, providing a suitable platform for further conductivity enhancement through po-Gr addition.

**Figure 1:**
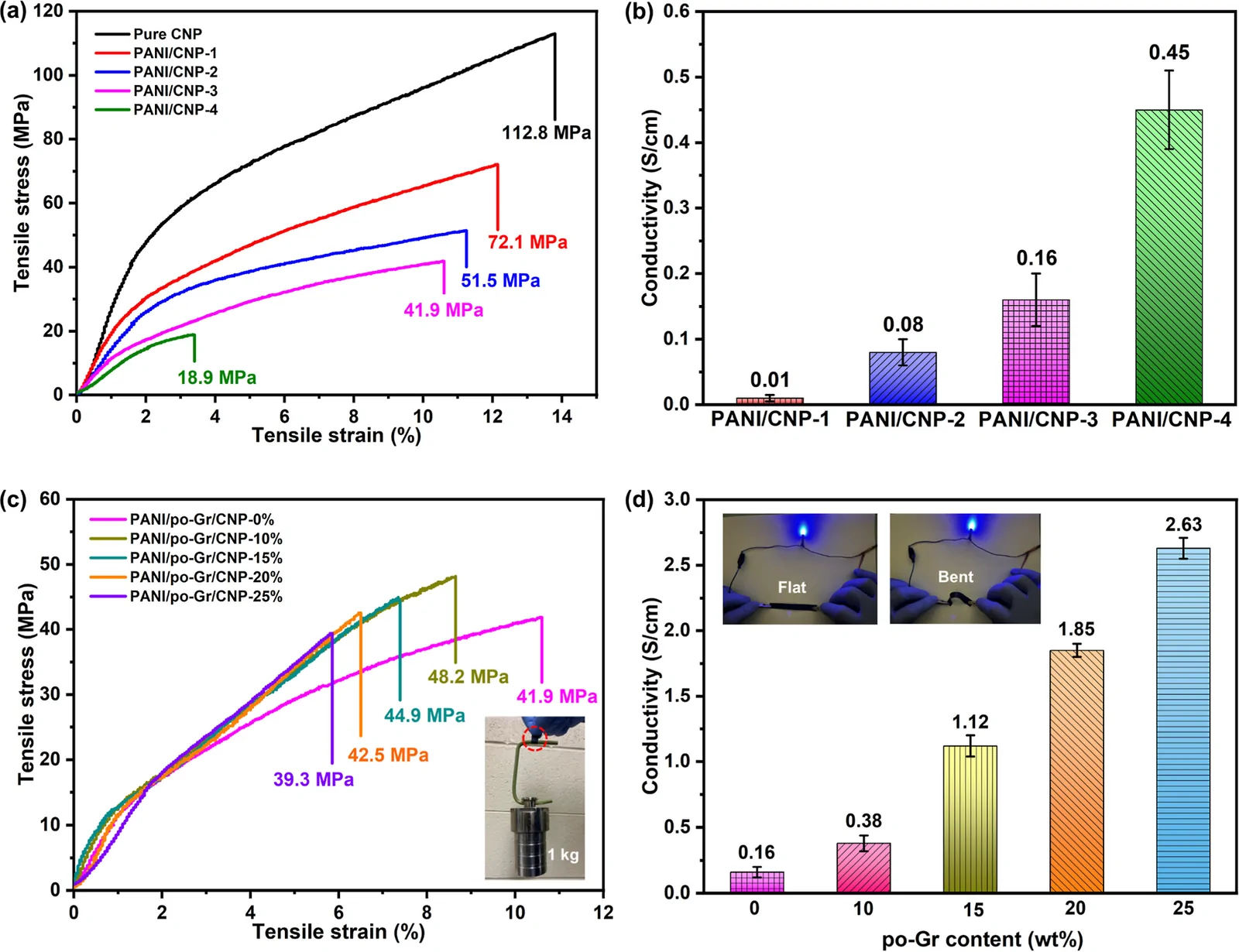
Stress–strain curves (a) and conductivity (b) of pure CNP and PANI/CNP samples with various PANI loadings (see Table 1 for details). Stress–strain curves (c) and electrical conductivity (d) of PANI/po-Gr/CNP samples with different po-Gr contents. The inset in (c) demonstrates the excellent load-bearing capability of the optimized PANI/po-Gr/CNP-20% film, which is able to support a 1 kg weight. Insets in (d) show that the optimized PANI/po-Gr/CNP film maintains electrical continuity in both flat and bent states.

To further improve charge transport while maintaining mechanical robustness, po-Gr was introduced into the optimized PANI/CNP-3 matrix. As shown in Figure 1c, incorporation of 10 wt % po-Gr increased the tensile strength from 41.9 to 48.2 MPa, indicating an effective reinforcing effect. The well-dispersed po-Gr nanosheets likely enhanced interfacial interactions and facilitated stress transfer throughout the hybrid network. However, further increasing the po-Gr content resulted in a gradual reduction in tensile strength. To further assess changes in film structure, we measured the thickness and apparent density of the films at different po-Gr loadings. As the po-Gr loading increased, the film thickness progressively decreased from 102 to 76 μm, while the apparent density, calculated from the dry film mass and dimensions, progressively increased from 0.755 to 1.013 g·cm^–3^. The tensile strength increased from 41.9 MPa for PANI/CNP to 48.2 MPa at 10 wt % po-Gr, but decreased to 42.5 MPa at 20 wt % po-Gr despite the continued increase in apparent density. This suggests that densification alone does not determine tensile strength. Changes in po-Gr distribution or the continuity of the CNF-based network may also affect stress transfer at higher loadings. In contrast to the relatively small changes in mechanical properties, po-Gr incorporation produced a remarkable enhancement in electrical conductivity (Figure 1d). The conductivity increased from 0.16 S·cm^–1^ to 0.38, 1.12, 1.85, and 2.63 S·cm^–1^ at po-Gr loadings of 10, 15, 20, and 25 wt %, respectively. The substantial increase originates from the formation of continuous conductive pathways by po-Gr nanosheets, which effectively bridge PANI-rich domains and reduce electron-transport resistance throughout the composite structure.

Although the film containing 25 wt % po-Gr exhibited the highest conductivity, its reduced tensile strength indicates compromised mechanical robustness. In comparison, the film containing 20 wt % po-Gr maintained a favorable tensile strength of 42.5 MPa while achieving a conductivity of 1.85 S·cm^–1^, representing more than an order-of-magnitude improvement relative to the po-Gr-free film. As further demonstrated by the inset in Figure 1c, the PANI/po-Gr/CNP-20% film was able to support a 1 kg weight without failure, highlighting its excellent mechanical robustness. In addition, the insets in Figure 1d show that the optimized PANI/po-Gr/CNP-20% film can complete an electrical circuit and illuminate an LED under both flat and bent conditions, demonstrating its good electrical conductivity and flexibility. Therefore, the PANI/po-Gr/CNP-20% film was identified as the optimal composition, providing the best balance between mechanical strength and electrical conductivity for flexible supercapacitor applications. This optimized composition was selected for subsequent characterization and electrochemical evaluation, and is hereafter denoted as PANI/po-Gr/CNP.

### 3.2 Surface Chemistry Characterization

The FTIR spectra of CNP, PANI, po-Gr, and their corresponding composites provide clear evidence for the successful formation of the hybrid structures (Figure 2a). The pure CNP exhibits the characteristic absorption bands of cellulose, including the broad O–H stretching vibration at approximately 3335 cm^–1^, C–H stretching vibrations at 2920–2850 cm^–1^, and C–O–C/C–O stretching vibrations in the range of 1062–980 cm^–1^, which are typical features of cellulose I.^37,38^ In contrast, pure PANI displays the characteristic bands of the emeraldine salt form, including the quinoid and benzenoid ring stretching vibrations at 1560 and 1480 cm^–1^, respectively, C–N stretching vibrations at 1300 and 1240 cm^–1^, and the characteristic electronic-like band at approximately 1140 cm^–1^ associated with protonated conductive PANI.^39,40^ For the PANI/CNP composite, characteristic bands from both cellulose and PANI are clearly observed, confirming the in situ polymerization of PANI on the CNF network. In addition, the O–H stretching band becomes weaker and shifts slightly toward lower wavenumbers compared with pure CNP, suggesting the formation of intermolecular interactions between CNFs and PANI and partial disruption of the original cellulose hydrogen-bonding network.^41^ This observation is consistent with the reduction in tensile strength observed after PANI incorporation.

**Figure 2:**
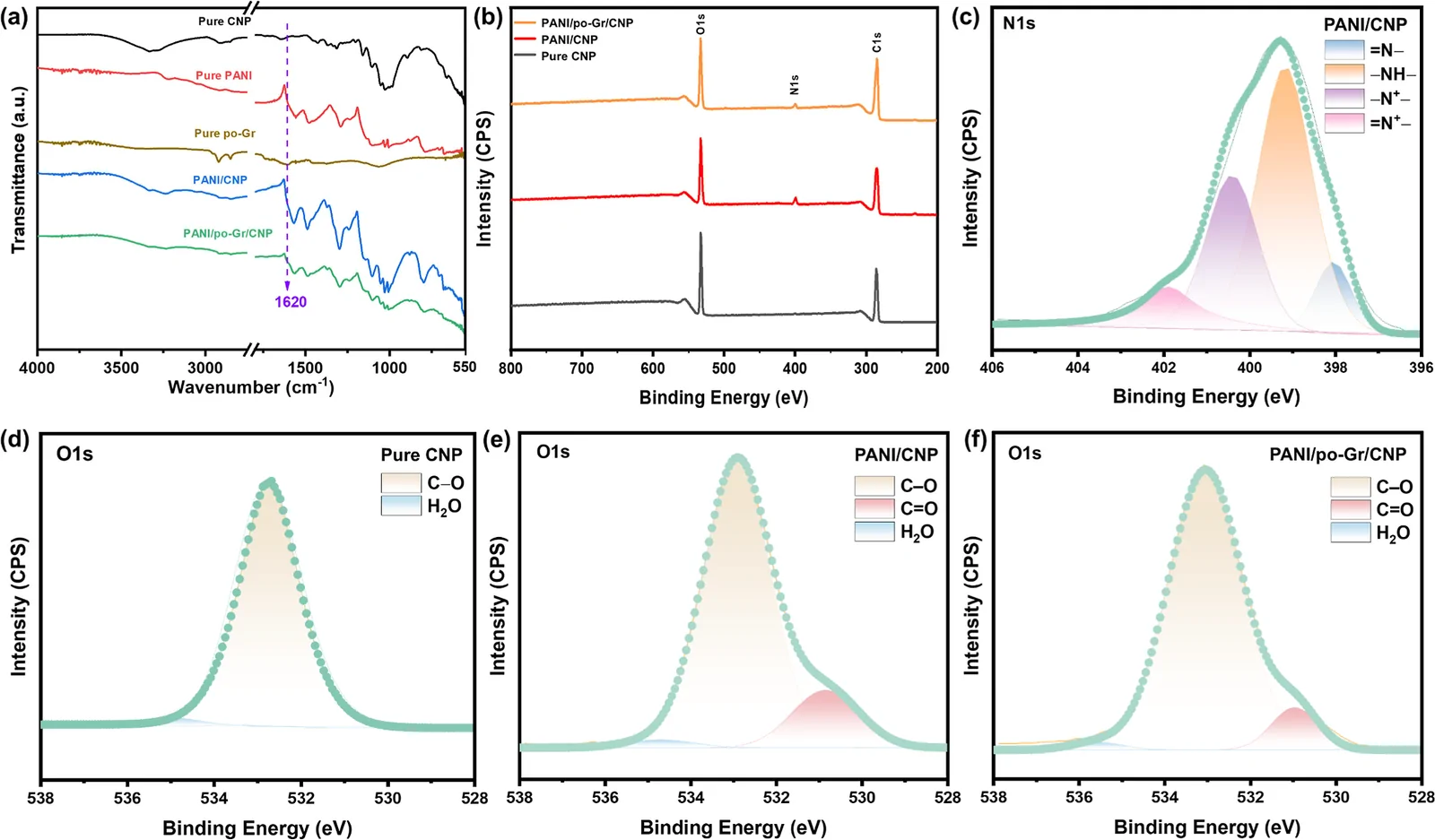
FTIR spectra of pure CNP, pure PANI, pure po-Gr, PANI/CNP, and PANI/po-Gr/CNP (a), XPS survey spectra of pure CNP, PANI/CNP, and PANI/po-Gr/CNP (b), High resolution N 1s spectra of the PANI/CNP (c), High resolution O 1s spectra of the pure CNP, PANI/CNP, and PANI/po-Gr/CNP (d–f).

The FTIR spectrum of po-Gr exhibits characteristic absorption bands at 1735 and 1706 cm^–1^ corresponding to carbonyl and carboxyl C=O stretching vibrations, together with a band at 1620 cm^–1^ assigned to the stretching vibration of sp^2^-hybridized graphitic C=C bonds.^23^ These features indicate that po-Gr retains graphitic domains while containing oxygen-containing functional groups introduced during partial oxidation. In the PANI/po-Gr/CNP composite, a weak but distinguishable band appears near 1620 cm^–1^, which can be attributed to the graphitic C=C vibration of po-Gr. The coexistence of characteristic cellulose, PANI, and po-Gr bands confirms the successful assembly of the ternary hybrid structure.

XPS analysis was further performed to investigate the surface elemental composition and chemical states of the composite films. As shown in Figure 2b, pure CNP displays only C 1s and O 1s signals, whereas an additional N 1s peak appears in the PANI/CNP sample, confirming the successful incorporation of nitrogen-containing PANI onto the CNFs. The PANI/po-Gr/CNP composite retains prominent C 1s, O 1s, and N 1s signals, indicating the coexistence of CNFs, PANI, and po-Gr within the hybrid structure. The high-resolution N 1s spectrum of PANI/CNP (Figure 2c) can be deconvoluted into four components corresponding to quinonoid imine (=N–, 398.2 eV), benzenoid amine (−NH–, 399.2 eV), protonated amine (−N^+^–, 400.5 eV), and protonated imine (=N^+^–, 402.0 eV).^42^ The presence of protonated nitrogen species confirms that PANI exists predominantly in its conductive emeraldine salt state, which is favorable for electron transport. This observation is also consistent with the characteristic FTIR band at approximately 1140 cm^–1^, further confirming the protonated structure of conductive PANI.

The high-resolution O 1s spectra in Figure 2d–f were fitted with components assigned to C–O, C=O, and adsorbed water, as applicable. Pure CNP is dominated by the C–O component associated with cellulose, whereas a C=O component is observed in both PANI/CNP and PANI/po-Gr/CNP. Thus, the C=O signal cannot be attributed solely to po-Gr incorporation. Together with the FTIR results, the XPS spectra are consistent with the expected chemical features of the composite films, but do not establish whether the components are chemically bonded or physically associated at their interfaces. The film morphology and the functional contributions of each component are examined in the following sections.

### 3.3 Morphology Characterization

The nanoscale morphology of the CNF and PANI/CNF suspensions was first investigated by TEM (Figure 3). As shown in Figure 3a, pristine CNFs exhibit long, flexible fibrils and fibril bundles with diameters in the nanometer range, forming a highly entangled network structure. Following in situ polymerization, numerous granular nanoparticles appear on the CNF surfaces (Figure 3b), indicating the successful deposition of PANI along the nanofiber network. The intimate contact between PANI and CNFs suggests that the CNFs serve as effective templates for the growth and dispersion of conductive PANI domains. To further verify the distribution of PANI, HAADF-STEM elemental mapping was performed (Figure 3c–f). Carbon and oxygen signals are uniformly distributed throughout the nanofiber network, consistent with the cellulose framework. More importantly, nitrogen and sulfur signals, originating from PANI and its dopant species, are homogeneously distributed along the fibrils, confirming the successful coating of CNFs with conductive PANI. The uniform distribution of PANI is expected to facilitate efficient electron transport while maintaining the interconnected nanofibrous architecture.

**Figure 3:**
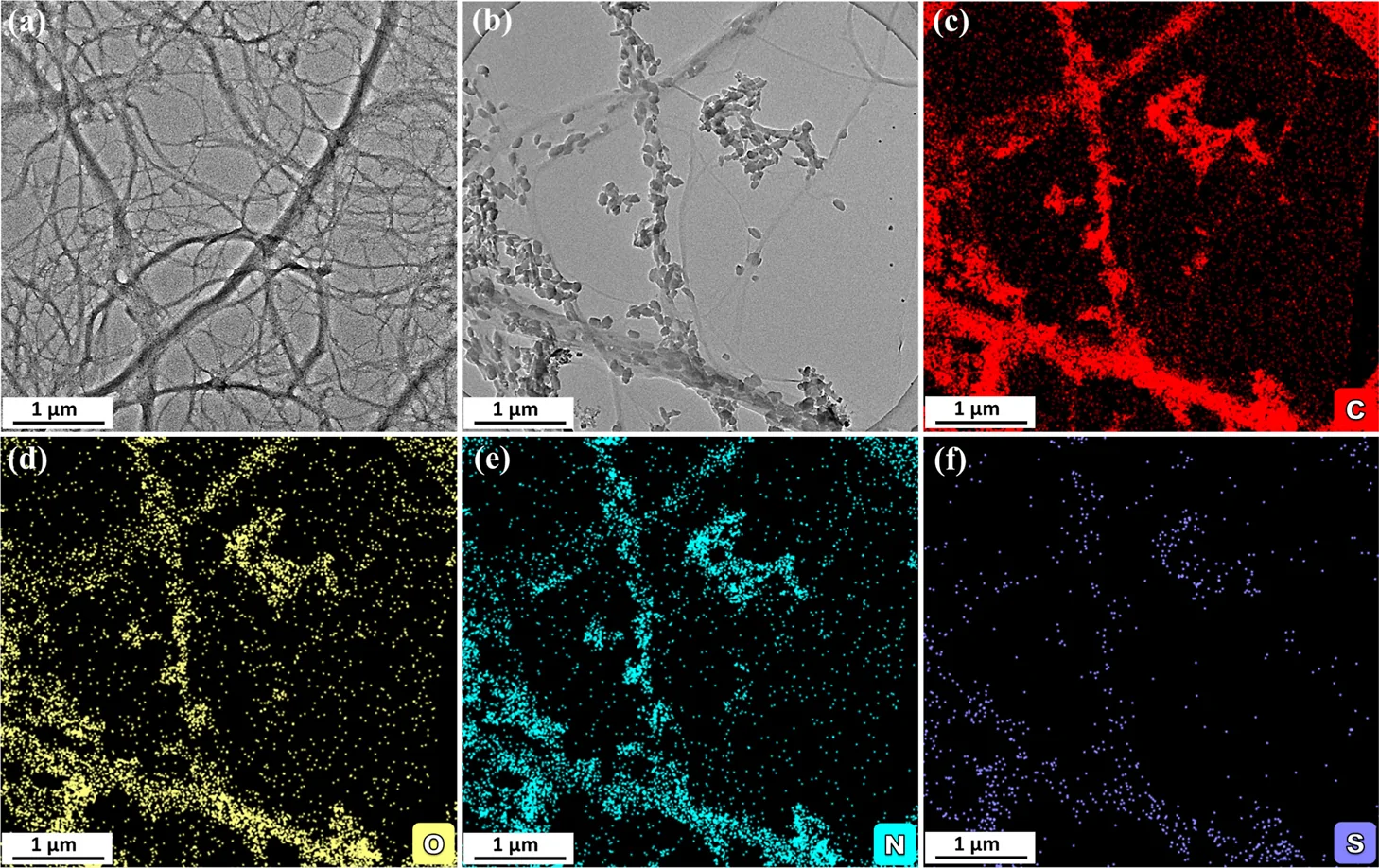
TEM images of CNF (a) and PANI/CNF (b). HAADF-STEM images of PANI/CNF and the corresponding elemental mappings of C, O, N, and S (c–f)

The morphology of the corresponding freestanding films was further examined by SEM (Figure 4). The pure CNP exhibits a dense and highly entangled layered structure composed of tightly packed nanofibers (Figure 4a). Such compact architecture is attributed to extensive hydrogen bonding among CNFs and is responsible for the excellent mechanical strength of the pristine nanopaper. After incorporation of PANI, the film morphology changes significantly (Figure 4b). The surface becomes noticeably rougher, and aggregated PANI particles are observed throughout the nanofiber network. The cross-sectional images reveal a less compact structure with increased interfibrillar voids, suggesting that PANI deposition partially disrupts the original hydrogen-bonding interactions between CNFs. This structural change is consistent with the decrease in tensile strength observed for PANI/CNP films, although the introduction of conductive PANI domains substantially improves the electrical conductivity.

**Figure 4:**
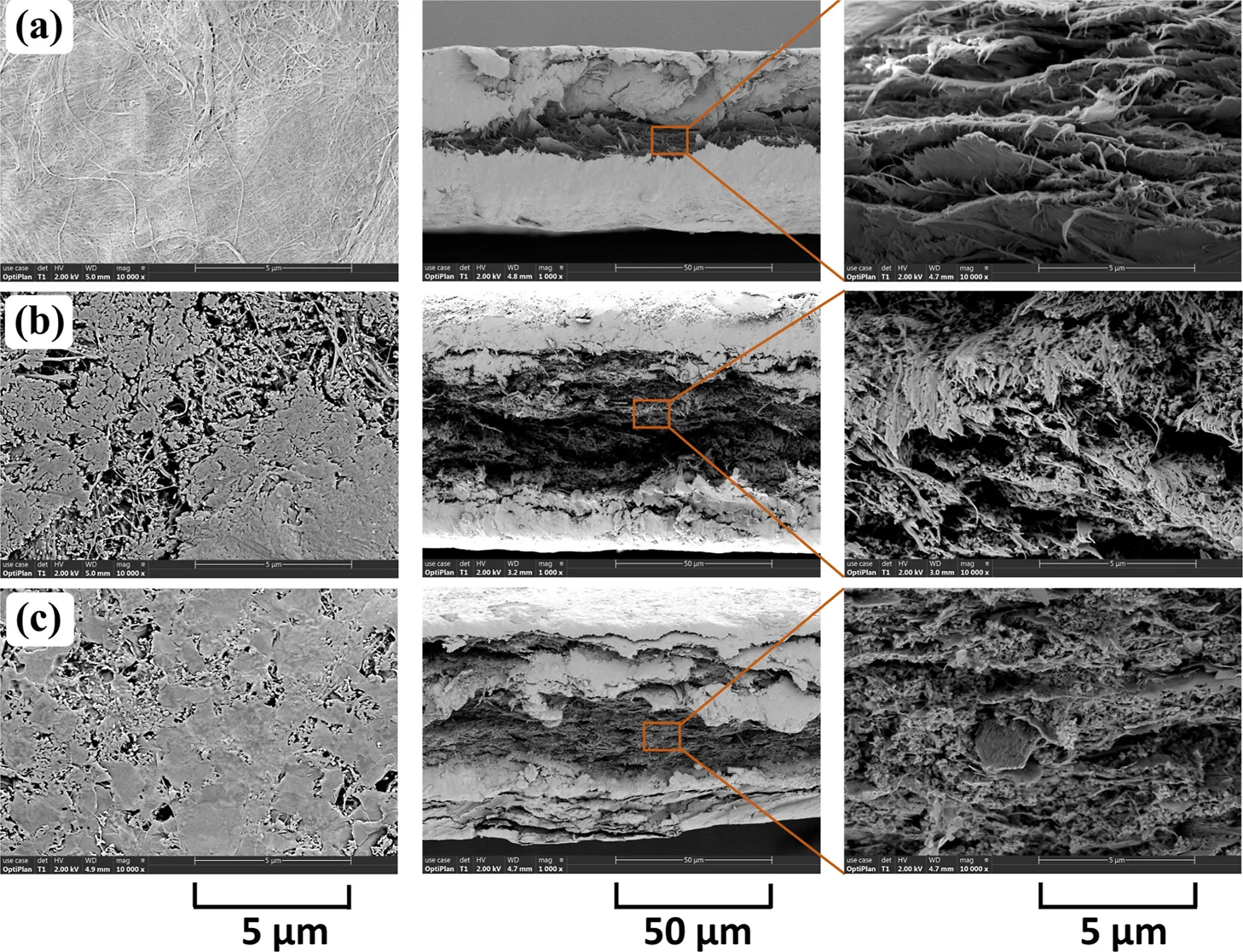
Surface and cross-sectional SEM images of CNP (a), PANI/CNP (b) and PANI/po-Gr/CNP (c)

A more integrated hierarchical structure is observed after incorporation of po-Gr (Figure 4c). Po-Gr nanosheets are distributed throughout the PANI/CNF matrix and can be clearly identified on both the film surface and cross-section. These nanosheets bridge adjacent PANI-coated nanofibers and create an interconnected conductive network within the layered architecture. Compared with the PANI/CNP film, the PANI/po-Gr/CNP composite exhibits a more continuous and compact structure, indicating enhanced interfacial interactions among po-Gr, PANI, and CNFs. Such multiscale architecture combines the mechanical support provided by the CNF framework, the pseudocapacitive functionality of PANI, and the highly conductive pathways introduced by po-Gr. The resulting interconnected network is expected to promote both electron transport and electrolyte ion accessibility, thereby contributing to the superior electrochemical performance of the freestanding electrode.

### 3.4 Electrochemical Performance of PANI/Po-Gr/CNP Electrodes

The electrochemical behavior of the PANI/po-Gr/CNP composites was first evaluated in a three-electrode configuration to elucidate the influence of po-Gr on charge-storage performance As shown in Figure 5a, all electrodes exhibit broad and nearly symmetric redox peaks, characteristic of the pseudocapacitive behavior of PANI. The incorporation of po-Gr does not alter the intrinsic redox mechanism of PANI. However, the CV curves gradually become larger and more rectangular with increasing po-Gr content, indicating enhanced charge-storage capability and reduced electrochemical polarization. This improvement can be attributed to the formation of more efficient electron-transport pathways, where po-Gr nanosheets bridge adjacent PANI-coated CNFs and facilitate rapid charge transfer throughout the electrode.

**Figure 5:**
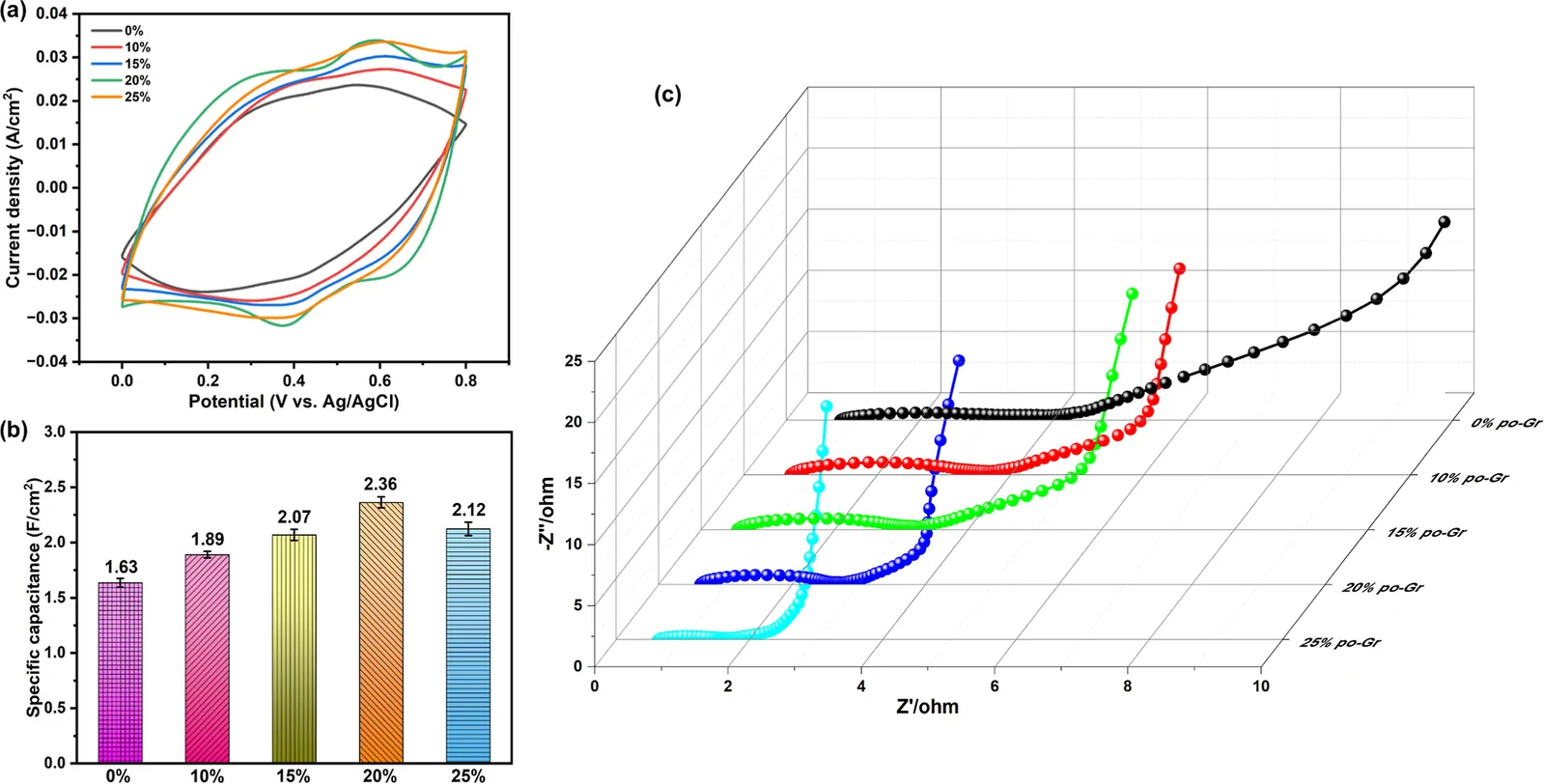
Electrochemical performance of PANI/po-Gr/CNP electrodes with varying po-Gr contents: (a) CV curves measured at 20 mV·s^–1^, (b) corresponding areal specific capacitances, and (c) Nyquist plots derived from EIS measurements.

The corresponding areal capacitances derived from the CV curves are summarized in Figure 5b. With increasing po-Gr content, the areal capacitance increases from 1.63 F·cm^–2^ for the po-Gr-free PANI/CNP electrode to 1.89, 2.07, and 2.36 F·cm^–2^ at po-Gr loadings of 10, 15, and 20 wt %, respectively. The enhancement is attributed to the highly conductive po-Gr network, which improves electrical connectivity between PANI domains and promotes more effective utilization of electroactive sites. When the po-Gr content was further increased to 25 wt %, the areal capacitance decreased slightly despite the improved electrical conductivity. This behavior can be attributed to two factors. First, excessive po-Gr loading may induce partial nanosheet aggregation, reducing ion accessibility to electroactive sites. Second, increasing the po-Gr fraction inevitably decreases the relative content of pseudocapacitive PANI within the electrode, thereby lowering the overall redox contribution to charge storage. As a result, further increasing the po-Gr content beyond 20 wt % does not lead to additional capacitance enhancement.

Electrochemical impedance spectroscopy (EIS) was performed to further investigate the charge-transfer characteristics of the electrodes (Figure 5c). With increasing po-Gr content, both the equivalent series resistance (R_s_) and charge-transfer resistance (R_ct_) gradually decrease, confirming the beneficial role of po-Gr in improving electrical conductivity and interfacial charge-transfer kinetics. In addition, the low-frequency region becomes progressively steeper, indicating enhanced capacitive behavior and more efficient ion diffusion within the electrode. Although the electrode containing 25 wt % po-Gr exhibits the lowest impedance, its reduced capacitance and inferior mechanical properties suggest that excessive po-Gr loading compromises overall electrode performance. In contrast, the 20 wt % po-Gr electrode combines high capacitance with low internal resistance, demonstrating a favorable balance between charge storage, electron transport, and structural stability.

The electrochemical performance of the optimized PANI/po-Gr/CNP electrode was further evaluated and compared with that of the po-Gr-free PANI/CNP electrode. As shown in Figure 6a, the CV curves recorded at scan rates ranging from 5 to 100 mV·s^–1^ exhibit well-defined and nearly symmetric redox peaks associated with the reversible doping/dedoping processes of PANI. Even at high scan rates, the CV profiles largely retain their shape, indicating rapid charge-transfer kinetics and favorable rate capability. Compared with the po-Gr-free electrode, the PANI/po-Gr/CNP electrode displays a substantially higher specific capacitance at all scan rates (Figure 6b), demonstrating enhanced charge-storage capability resulting from the improved electrical conductivity and more efficient utilization of electroactive PANI sites.

**Figure 6:**
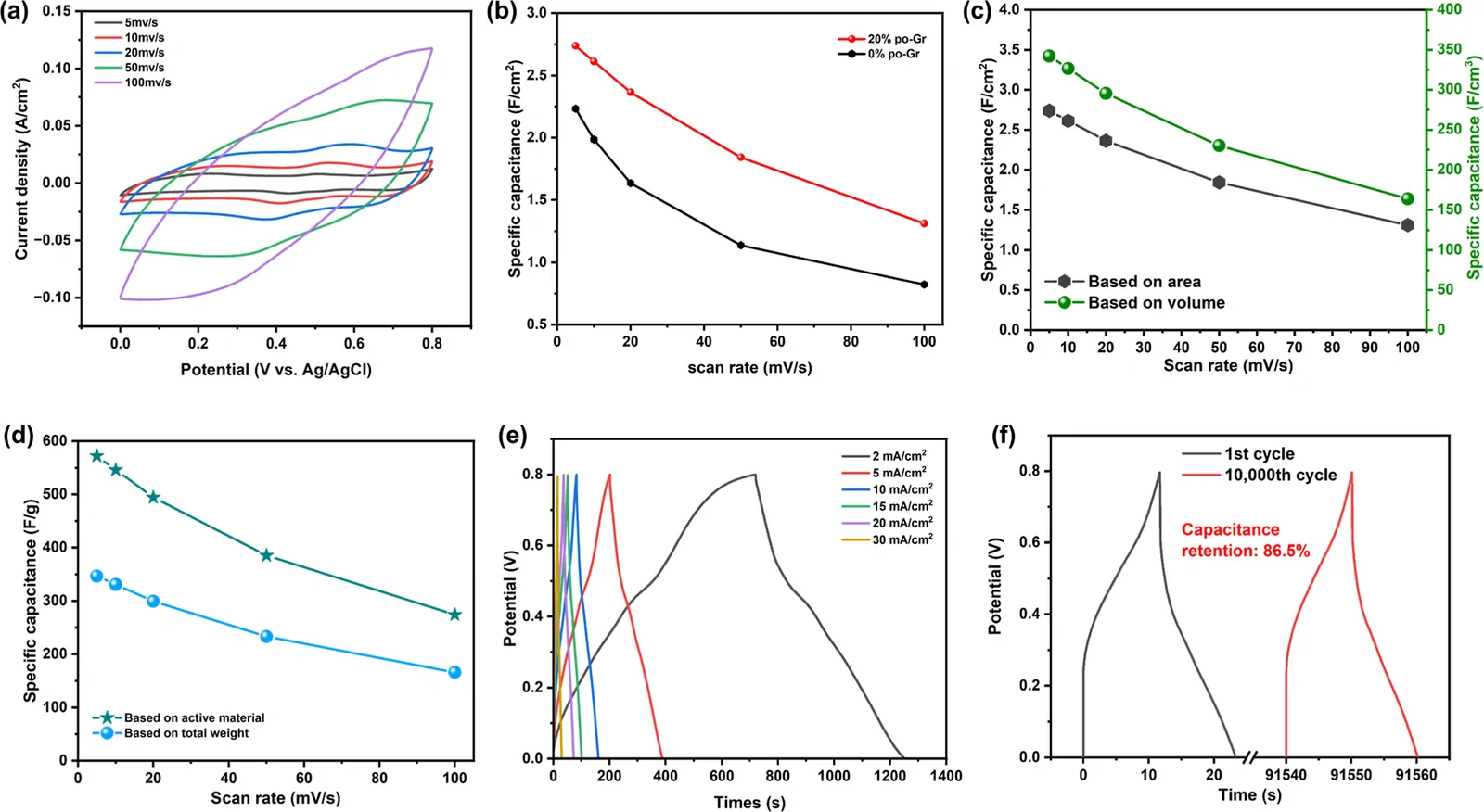
Electrochemical properties of the optimized PANI/po-Gr/CNP electrode in three-electrode systems: CV curves at different scan rates (a); specific areal capacitance compared with the PANI/CNP electrode (b); areal and volumetric specific capacitance (c); gravimetric specific capacitance based on active material and total weight of the electrode (d); GCD curves at different current densities (e); GCD curves at the 1st and 10,000th cycles at a current density of 50 mA·cm^–2^ (f).

The specific capacitances calculated on the basis of area, volume, and mass further highlight the excellent charge-storage capability of the optimized electrode. As shown in Figure 6c and d and summarized in Table 2, the PANI/po-Gr/CNP electrode achieves an areal capacitance of 2.74 F·cm^–2^, a volumetric capacitance of 342.5 F·cm^–3^, and a gravimetric capacitance of 572.7 F·g^–1^ based on the active materials at 5 mV·s^–1^. When normalized to the total electrode mass, a gravimetric capacitance of 345.2 F·g^–1^ is obtained. The high volumetric capacitance originates from the compact layered nanopaper architecture, while the high gravimetric capacitance benefits from the efficient integration of conductive po-Gr nanosheets and redox-active PANI within the CNF framework. Notably, these electrochemical properties are achieved while maintaining a tensile strength of 42.5 MPa, highlighting the ability of the freestanding nanopaper electrode to simultaneously provide high electrochemical performance and mechanical robustness.

**Table 2:** Comparison of PANI/Po-Gr/CNP Electrode With Other PANI-Based Electrode Materials^a^

material	capacitance	No. cycles, stability (%)	mechanical strength (MPa)	ref
PANI: PSS/CNF	233 F/g at 2 mA/cm^2^	8000, 81.5	29.1	^41^
BC/PANI	273 F/g at 0.2 A/g	1000, 94.3		^43^
PANI/Gr/BC	4.16 F/cm^2^ at 1 mA/cm^2^	2000, 91.5		^44^
PANI/Ag/CNF	0.176 F/cm^2^ at 10 mV/s			^45^
CNF/MWCNT/PANI	249.7 F/g at 10 mV/s	1000, 82.4		^46^
Cellulose fiber/SWCNT/PANI	0.33 F/cm^2^ at 2 mA/cm^2^			^47^
MXene/CNF/PANI	2.94 F/cm^2^ at 1 mA/cm^2^	4000, 81.5		^48^
TOCN/SCNT/PANI	1.79 F/cm^2^ at 1 mA/cm^2^	1000, 85.22		^49^
Cellulose/GO/PANI	1.22 F/cm^2^ at 1 mA/cm^2^			^50^
CMC-PANI/CNT	3.11 F/cm^2^ at 5 mA/cm^2^	5000, 89.2		^19^
TOCNF/CNT/PANI	2.18 F/cm^2^ at 1 mA/cm^2^	1050, 64		^51^
MXene/CMC-PANI	1.16 F/cm^2^ at 1 mA/cm^2^		35.6	^52^
NC/PANI/RGO	79.71 F/g at 0.2 A/g		8.83	^53^
BC/PANI/Mxene	337 F/g at 1 A/g	10,000, 84.1		^54^
PANI/DamCNC	240 F/g at 1 A/g			^55^
**This work**	345.2 F/g based on whole film at 5 mV/s	10,000, 86.5	42.5	
	572.7 F/g based on active material at 5 mV/s			
	2.74 F/cm^2^ at 5 mV/s			
	342.5 F/cm^3^ at 5 mV/s			

a The gravimetric capacitances of 345.2 and 572.7 F·g^–1^ are normalized by the total dry mass of the PANI/po-Gr/CNP electrode and the combined mass of PANI and po-Gr, respectively. The PANI content in the PANI/CNP precursor was approximately 50.7 wt % before po-Gr incorporation. The areal and volumetric capacitances are normalized by the electrode area and volume, respectively. All four values were determined at a scan rate of 5 mV·s^–1^.

The GCD curves measured at different current densities (Figure 6e) exhibit nearly symmetric charge and discharge profiles, indicating high reversibility and good capacitive behavior. The relatively small IR drop observed for the PANI/po-Gr/CNP electrode is consistent with its enhanced electrical conductivity and reduced internal resistance, as evidenced by the EIS results. Furthermore, the charge–discharge profiles remain well preserved even at high current densities, demonstrating efficient charge transport throughout the electrode. Long-term cycling stability was evaluated at a current density of 50 mA·cm^–2^ (Figure 6f). After 10,000 charge–discharge cycles, the electrode retains 86.5% of its initial capacitance, while the GCD profile remains nearly unchanged. The improved electrochemical performance and cycling stability can be understood from the complementary roles of the three components. The entangled CNF network provides mechanical support for the freestanding electrode, while PANI coated on the CNFs contributes redox-active sites for charge storage. The po-Gr nanosheets bridge neighboring PANI-coated fibers, forming additional electron-transport pathways, consistent with the increase in conductivity from 0.16 to 1.85 S·cm^–1^. The hybrid network may also help maintain electrical contact during repeated PANI doping and dedoping, contributing to the observed capacitance retention after 10,000 cycles.

### 3.5 Electrochemical Performance of Flexible Symmetric Supercapacitors

To further evaluate the practical applicability of the optimized PANI/po-Gr/CNP electrode, a symmetric supercapacitor was assembled using two identical PANI/po-Gr/CNP films and a cellulose separator soaked in 1.0 M H_2_SO_4_ electrolyte (Figure 7a). The CV curves recorded at scan rates ranging from 5 to 50 mV·s^–1^ exhibit quasi-rectangular shapes with broad redox humps (Figure 7b), indicating the coexistence of electric double-layer capacitance and pseudocapacitance originating from the PANI/po-Gr hybrid electrode. The CV profiles remain well preserved with increasing scan rate, demonstrating favorable rate capability and rapid charge-transfer kinetics within the device. The GCD curves measured at different current densities exhibit nearly symmetric triangular shapes with only a small IR drop (Figure 7c), indicating highly reversible charge-storage behavior and low internal resistance. As shown in Figure 7d and e, the assembled device delivers a maximum areal capacitance of 0.65 F·cm^–2^ and a volumetric capacitance of 82 F·cm^–3^ at 5 mV·s^–1^. In addition, gravimetric capacitances of 135 F·g^–1^ and 82 F·g^–1^ are achieved based on the active materials and total electrode mass, respectively. These results demonstrate the efficient utilization of electroactive PANI and the effective electron-transport pathways established by the po-Gr nanosheets within the freestanding nanopaper architecture.

**Figure 7:**
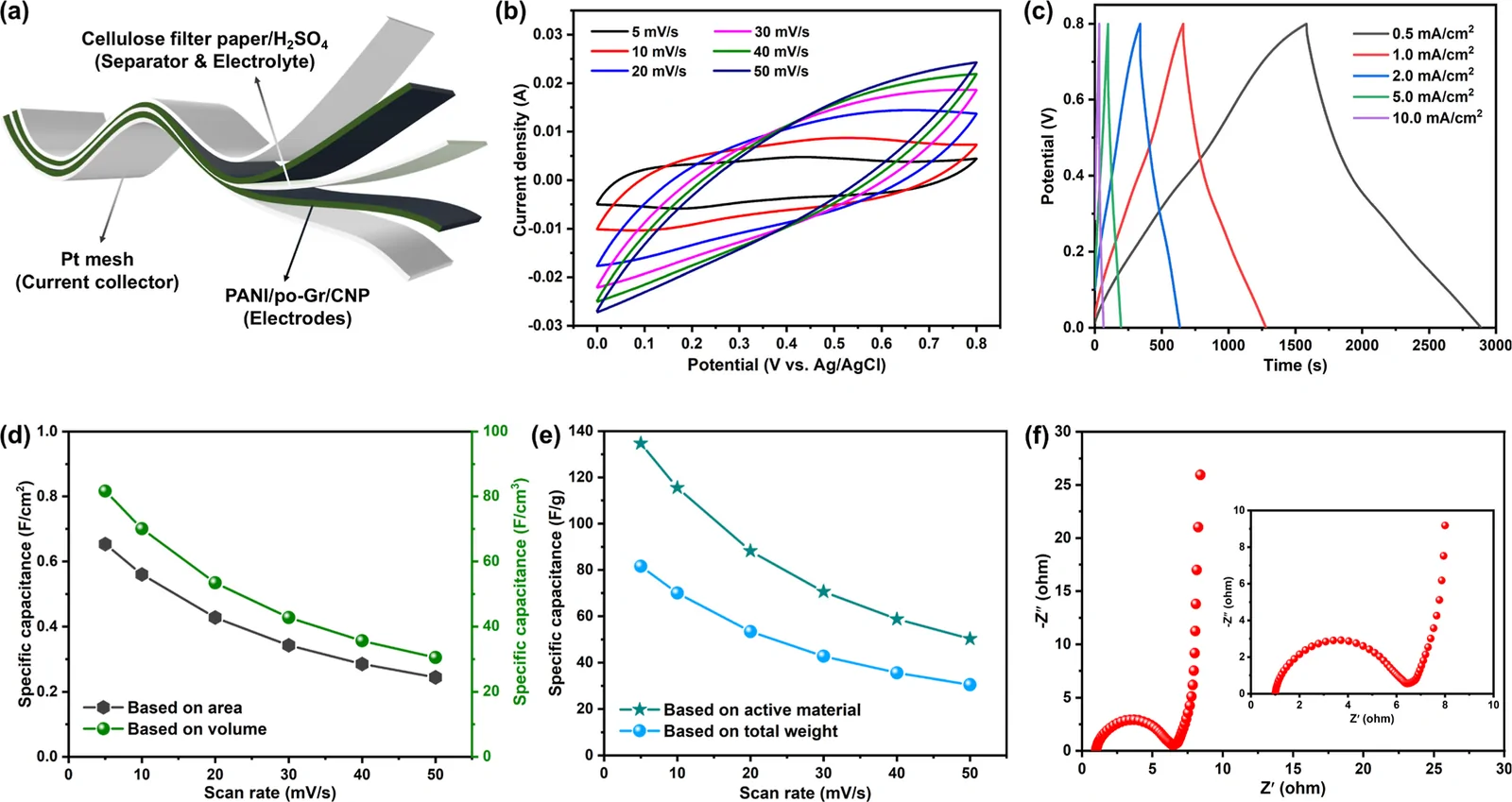
Schematic illustration of the assembled supercapacitor device (a). Electrochemical properties of the supercapacitor device in two-electrode systems: CV curves at different scan rates (b); GCD curves at different current densities (c); areal and volumetric specific capacitance at 5 mV·s^–1^ (d); gravimetric specific capacitance at 5 mV·s^–1^ based on active material and total weight of the electrodes (e), and Nyquist plot (f).

The impedance characteristics of the symmetric supercapacitor were further analyzed by EIS (Figure 7f). The Nyquist plot consists of a small high-frequency semicircle followed by a nearly vertical line in the low-frequency region, indicating low charge-transfer resistance and favorable capacitive behavior. As expected, both the R_s_ and Rct are slightly higher than those measured for the individual electrode in the three-electrode configuration because of the additional contributions from the second electrode, separator, electrolyte, and interfacial contacts. Nevertheless, the overall impedance remains low, confirming efficient ion diffusion and continuous electron-transport pathways throughout the assembled device.

Long-term cycling stability was further evaluated at a current density of 10 mA·cm^–2^ (Figure 8a). After 10,000 charge–discharge cycles, the device retains 81.0% of its initial capacitance, while the GCD profiles remain nearly unchanged, demonstrating good electrochemical cycling stability under the tested conditions. The energy storage capability of the assembled device was further assessed using a Ragone plot (Figure 8b). The symmetric supercapacitor delivers an areal energy density of 58.08 μWh·cm^–2^ at a power density of 1306.8 μW·cm^–2^ and a volumetric energy density of 7.26 mWh·cm^–3^ at 163.35 mW·cm^–3^. When normalized to the total electrode mass, the device achieves an energy density of 7.35 Wh·kg^–1^ at a power density of 165.42 W·kg^–1^. These values are comparable to, and in some cases exceed, those reported for representative PANI/cellulose-based supercapacitors in the literature, highlighting the effectiveness of the hierarchical PANI/po-Gr/CNP architecture in simultaneously achieving high areal, volumetric, and gravimetric energy-storage performance.

**Figure 8:**
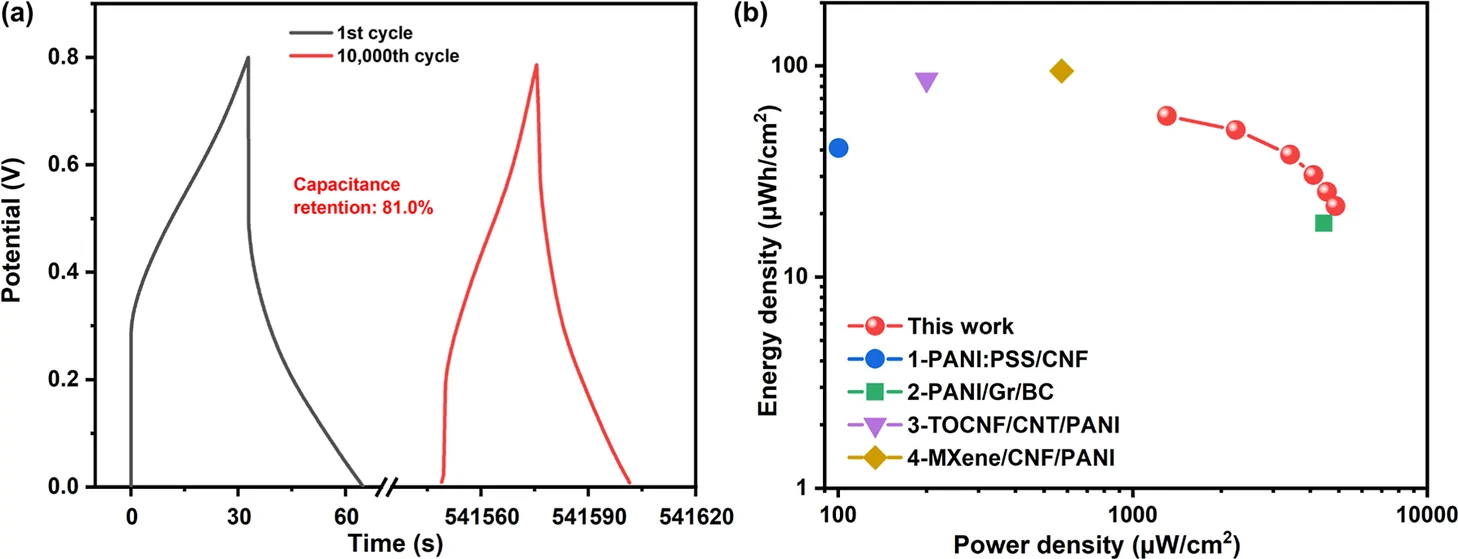
(A) GCD curves of the supercapacitor device at the first and 10,000th cycles (at a current density of 10 mA·cm^–2^). (b) Areal Ragone plot comparing the PANI/po-Gr/CNP supercapacitor developed in this work with previously reported PANI/cellulose composite electrodes. 1-PANI:PSS/CNF,^41^ 2-PANI/Gr/BC,^44^ 3-TOCNF/CNT/PANI,^51^ and 4-MXene/CNF/PANI.^48^

The practical applicability of the assembled PANI/po-Gr/CNP supercapacitors was further evaluated through series and parallel integration. As shown in Figure 9a and b, connecting multiple devices in series increased the operating voltage window proportionally from 0.8 to 2.4 V. At the same time, the discharge time remained nearly unchanged under the same current density. This behavior is consistent with the theoretical characteristics of capacitors connected in series, where the overall capacitance decreases while the output voltage increases. In contrast, parallel connection of the devices (Figure 9c,d) resulted in progressively higher current responses and longer discharge times without changing the operating voltage window, indicating an increase in the overall capacitance and charge-storage capability of the integrated system. In both series and parallel configurations, the CV and GCD curves retained their characteristic shapes without noticeable distortion, demonstrating excellent electrochemical reversibility, stable device performance, and reliable electrical connections between individual cells. To further demonstrate their practical potential, three supercapacitor devices connected in series were used to power a digital timer (Figure 9e,f). The timer operated continuously for at least 10 min without interruption, confirming the ability of the assembled devices to deliver stable power output for low-power electronics.

**Figure 9:**
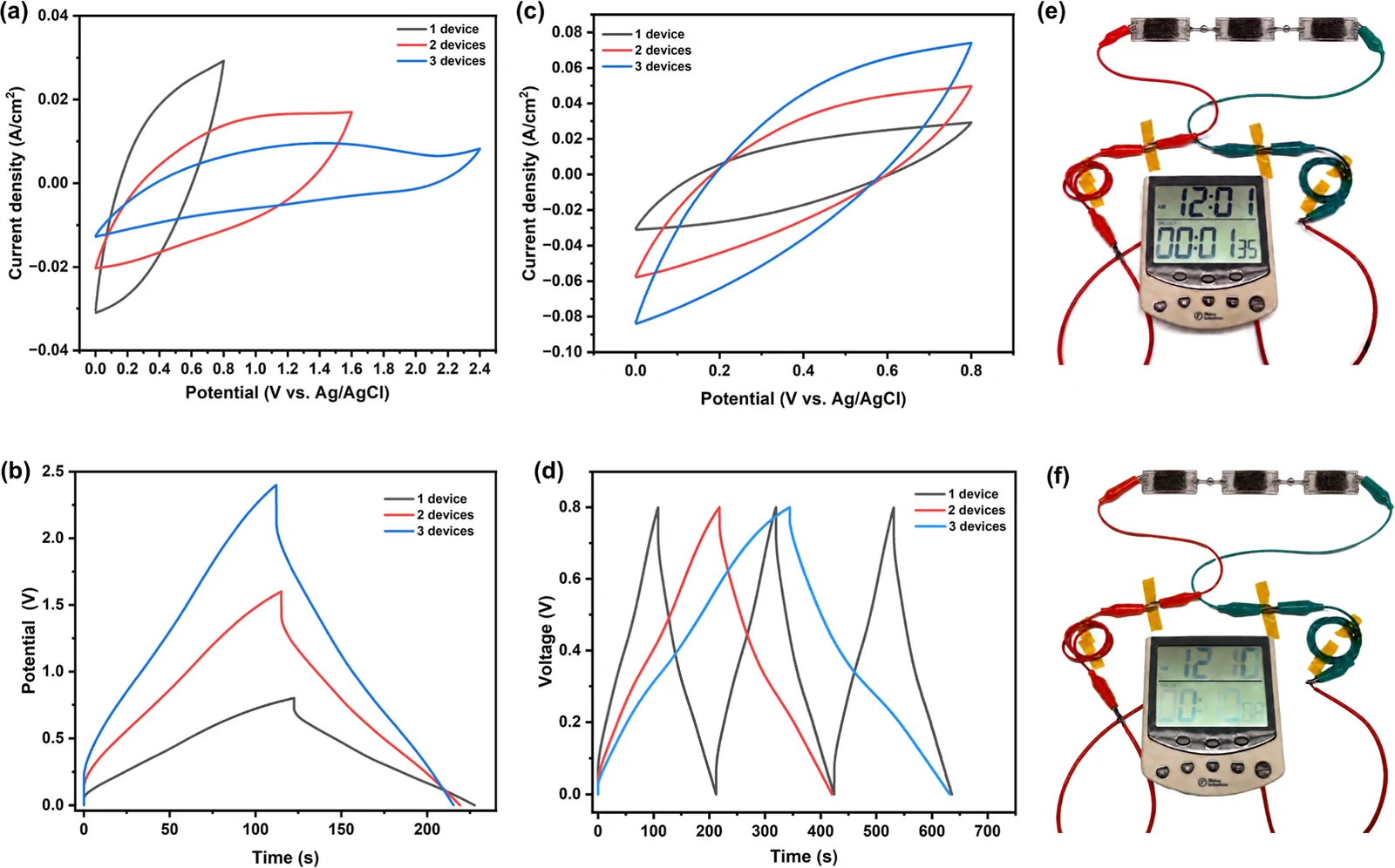
Series and parallel integration of PANI/po-Gr/CNP symmetric supercapacitors. (a, b) CV curves (50 mV·s^–1^) and GCD curves (5 mA·cm^–2^) of one, two, and three devices connected in series. (c, d) CV curves (50 mV·s^–1^) and GCD curves (5 mA·cm^–2^) of one, two, and three devices connected in parallel. (e, f) Demonstration of a digital timer powered by three devices connected in series after 1 and 10 min, respectively.

Overall, the excellent capacitive behavior, low internal resistance, good rate capability, and long-term cycling stability demonstrate that the PANI/po-Gr/CNP electrode can be readily translated from an individual electrode to a practical device configuration. The integration of mechanically robust CNFs, redox-active PANI, and conductive po-Gr nanosheets provides an effective strategy for developing high-performance freestanding supercapacitors for flexible and wearable energy storage applications.

## 4 Conclusions

In summary, a flexible and freestanding PANI/po-Gr/CNP composite electrode was developed through the integration of CNFs, PANI, and po-Gr. The CNFs provided a mechanically robust and flexible framework, while the uniformly distributed PANI coating supplied abundant redox-active sites, and the po-Gr nanosheets established efficient electron-transport pathways throughout the electrode. By systematically optimizing the PANI and po-Gr contents, an excellent balance among mechanical strength, electrical conductivity, and electrochemical performance was achieved. The resulting electrode delivered a high areal capacitance of 2.74 F·cm^–2^ at 5 mV·s^–1^, with gravimetric capacitances of 345.2 F·g^–1^ based on the total dry electrode mass and 572.7 F·g^–1^ based on the combined mass of PANI and po-Gr. It retained 86.5% of its initial capacitance after 10,000 charge–discharge cycles, demonstrating outstanding long-term cycling stability. When assembled into a symmetric supercapacitor, the device achieved high areal, volumetric, and gravimetric energy storage performance together with good electrochemical cycling stability. Furthermore, the assembled devices could be readily connected in series and parallel to tailor the output voltage and capacitance. The hierarchical PANI/po-Gr/CNP architecture provides an effective strategy for simultaneously enhancing electron transport, ion accessibility, and structural stability, offering a promising platform for flexible and wearable energy storage devices.

## Data Availability

The data that support the findings of this study are available from the corresponding authors upon reasonable request.

## References

[1] Nadeem F., Hussain S. S., Tiwari P. K., Goswami A. K., Ustun T. S. (2019). Comparative
review of energy storage systems, their roles, and impacts on future
power systems. IEEE Access.

[2] Chen X., Paul R., Dai L. (2017). Carbon-based supercapacitors
for
efficient energy storage. Natl. Sci. Rev..

[3] Liu J., Wang J., Xu C., Jiang H., Li C., Zhang L., Lin J., Shen Z. X. (2018). Advanced Energy
Storage Devices: Basic Principles, Analytical Methods, and Rational
Materials Design. Adv. Sci. (Weinh).

[4] Sharma P., Bhatti T. (2010). A review on electrochemical double-layer
capacitors. Energy Convers. Manage..

[5] Schütter C., Pohlmann S., Balducci A. (2019). Industrial
requirements of materials
for electrical double layer capacitors: impact on current and future
applications. Adv. Energy Mater..

[6] Fleischmann S., Mitchell J. B., Wang R., Zhan C., Jiang D. E., Presser V., Augustyn V. (2020). Pseudocapacitance:
From Fundamental
Understanding to High Power Energy Storage Materials. Chem. Rev..

[7] Alharbi A. (2025). Boosting supercapacitor
performance through innovative transition metal-based electrode materials. RSC Adv..

[8] Mandal S., Mendhe A. B., Rakhade H. M., Barse N. S., Roy M., Rosaiah P., Park T., Lee H.-S., Mendhe A. C., Kim D. (2025). Recent advancement and design in supercapacitor hybrid electrode
materials: Bridging the gap between energy and power density. Chem. Eng. J. Adv..

[9] Salaheldeen M., Eskander T. N. A., Fathalla M., Zhukova V., Blanco J. M., Gonzalez J., Zhukov A., Abu-Dief A. M. (2025). Empowering the Future:
Cutting-Edge Developments in Supercapacitor Technology for Enhanced
Energy Storage. Batteries.

[10] Liu W., Song M.-S., Kong B., Cui Y. (2017). Flexible and Stretchable
Energy Storage: Recent Advances and Future Perspectives. Adv. Mater..

[11] Benzigar M. R., Dasireddy V. D., Guan X., Wu T., Liu G. (2020). Advances on
emerging materials for flexible supercapacitors: current trends and
beyond. Adv. Funct. Mater..

[12] Keum K., Kim J. W., Hong S. Y., Son J. G., Lee S. S., Ha J. S. (2020). Flexible/stretchable supercapacitors with novel functionality for
wearable electronics. Adv. Mater..

[13] Kulandaivalu S., Sulaiman Y. (2019). Recent Advances in Layer-by-Layer
Assembled Conducting
Polymer Based Composites for Supercapacitors. Energies.

[14] I.
O O., S. O A., A. S T., I. M A., O. F O., A. O O. (2025). Polymer-based nanocomposites for supercapacitor applications: a review
on principles, production and products. RSC
Adv..

[15] Lizarraga L., María
Andrade E., Victor Molina F. (2004). Swelling and
volume changes of polyaniline upon redox switching. J. Electroanal. Chem..

[16] Liu J., Du P., Wang Q., Liu D., Liu P. (2019). Mild synthesis of holey
N-doped reduced graphene oxide and its double-edged effects in polyaniline
hybrids for supercapacitor application. Electrochim.
Acta.

[17] Hu N., Zhang L., Yang C., Zhao J., Yang Z., Wei H., Liao H., Feng Z., Fisher A., Zhang Y. (2016). Three-dimensional
skeleton networks of graphene wrapped polyaniline
nanofibers: an excellent structure for high-performance flexible solid-state
supercapacitors. Sci. Rep..

[18] Tian J., Cui N., Chen P., Guo K., Chen X. (2021). High-performance wearable
supercapacitors based on PANI/N-CNT@CNT fiber with a designed hierarchical
core-sheath structure. Journal of Materials
Chemistry A.

[19] Xu H., Cui L., Pan X., An Y., Jin X. (2022). Carboxymethylcellulose-polyaniline/carbon
nanotube (CMC-PANI/CNT) film as flexible and highly electrochemical
active electrode for supercapacitors. Int. J.
Biol. Macromol..

[20] Urade A. R., Lahiri I., Suresh K. (2023). Graphene properties,
synthesis and
applications: a review. JOM.

[21] Al
Faruque M. A., Syduzzaman M., Sarkar J., Bilisik K., Naebe M. (2021). A review on the production methods and applications of graphene-based
materials. Nanomaterials.

[22] Zhang F., Yang K., Liu G., Chen Y., Wang M., Li S., Li R. (2022). Recent advances on graphene: Synthesis, properties
and applications. Composites, Part A.

[23] Sadak O., Sundramoorthy A. K., Gunasekaran S. (2018). Facile and green synthesis of highly
conducting graphene paper. Carbon.

[24] Drummond R., Huang C., Grant P., Duncan S. (2019). Overcoming diffusion
limitations in supercapacitors using layered electrodes. J. Power Sources.

[25] Xu T., Du H., Liu H., Liu W., Zhang X., Si C., Liu P., Zhang K. (2021). Advanced Nanocellulose-Based
Composites for Flexible
Functional Energy Storage Devices. Adv. Mater..

[26] Liu H., Du H., Zheng T., Liu K., Ji X., Xu T., Zhang X., Si C. (2021). Cellulose
based composite foams and
aerogels for advanced energy storage devices. Chemical Engineering Journal.

[27] Ruan S., Zhu S., Dong Y., Gong R., Yu H.-Y. (2026). Ion Coordination–Reduction
Synergy Constructs CNFene/Cu Nanoparticles to Regenerate Silk Fibroin
Fibers for Reliable and Precise Smart Pulse Diagnosis. Adv. Fiber Mater..

[28] Dong Y., Ruan S., Li S., Zhu S., Ke Y., Jiang M., Tam K. C., Yu H. (2026). Skin-Inspired Janus
Architecture Self-Decoupled Dual-Module Sensing System for Smart Home
Health Management. Adv. Funct. Mater..

[29] Du H., Liu K., Xu T., Xu C., Lin M., Fang Z., Kim S. W., Seo J. Y., Chen J., Ma H. (2025). Lignocellulosic Films:
Preparation, Properties, and Applications. Chem.
Rev..

[30] Zhai W., Zhong Y., Zhang W., Ren Z., Ji T., Ding K., Chen S., Wei X., Cai L., Xia C. (2025). Biofunctional cellulose fibers from mulberry bast via
suberin nanointerface engineering. J. Bioresour.
Bioprod..

[31] Yang K., Duan C., Wen Y., Tian G., Ma R., Liu X., Bie Y. (2025). Hierarchically
porous coaxial wet-spun cellulose/polyurethane
based hexamethylene diisocyanate (PUH) solid-solid phase change fiber
for enhanced thermal management. J. Bioresour.
Bioprod..

[32] Shen J., Zhang M. (2025). Disassembly, refinement,
and reassembly: From ancient papermaking
to modern materials processing. J. Bioresour.
Bioprod..

[33] Liu W., Liu K., Du H., Zheng T., Zhang N., Xu T., Pang B., Zhang X., Si C., Zhang K. (2022). Cellulose
Nanopaper: Fabrication, Functionalization, and Applications. Nanomicro Lett..

[34] Zhao D., Zhu Y., Cheng W., Chen W., Wu Y., Yu H. (2021). Cellulose-Based
Flexible Functional Materials for Emerging Intelligent Electronics. Adv. Mater..

[35] Weng M., Zhou J., Ye Y., Qiu H., Zhou P., Luo Z., Guo Q. (2023). Self-chargeable supercapacitor made with MXene-bacterial
cellulose nanofiber composite for wearable devices. J. Colloid Interface Sci..

[36] Li X., Yuan L., Liu R., He H., Hao J., Lu Y., Wang Y., Liang G., Yuan G., Guo Z. (2021). Engineering
Textile Electrode and Bacterial Cellulose Nanofiber Reinforced Hydrogel
Electrolyte to Enable High-Performance Flexible All-Solid-State Supercapacitors. Adv. Energy Mater..

[37] Zhang Y., Deng W., Wu M., Yu G., Liu Z., Cheng N., Du H., Liu C., Li B. (2024). Engineering
pulp foam with highly improved water stability and multifunctional
properties by incorporation of natural rubber and montmorillonite. Green Carbon.

[38] Du H., Parit M., Wu M., Che X., Wang Y., Zhang M., Wang R., Zhang X., Jiang Z., Li B. (2020). Sustainable valorization of paper mill sludge into cellulose nanofibrils
and cellulose nanopaper. J. Hazard. Mater..

[39] Wu G., Du H., Lee D., Cha Y. L., Kim W., Zhang X., Kim D. J. (2022). Polyaniline/Graphene-Functionalized
Flexible Waste
Mask Sensors for Ammonia and Volatile Sulfur Compound Monitoring. ACS Appl. Mater. Interfaces.

[40] Wu G., Du H., Pakravan K., Kim W., Cha Y. L., Chiang S.-T., Beidaghi M., Zhang X., Kim S. H., Pan X. (2023). Polyaniline/Ti3C2Tx functionalized mask sensors for
monitoring of
CO2 and human respiration rate. Chemical Engineering
Journal.

[41] Liang Y., Wei Z., Wang H.-E., Flores M., Wang R., Zhang X. (2022). Flexible and
freestanding PANI: PSS/CNF nanopaper electrodes with enhanced electrochemical
performance for supercapacitors. J. Power Sources.

[42] Zhang M., Nautiyal A., Du H., Wei Z., Zhang X., Wang R. (2021). Electropolymerization of polyaniline
as high-performance binder free
electrodes for flexible supercapacitor. Electrochim.
Acta.

[43] Wang H., Zhu E., Yang J., Zhou P., Sun D., Tang W. (2012). Bacterial
Cellulose Nanofiber-Supported Polyaniline Nanocomposites with Flake-Shaped
Morphology as Supercapacitor Electrodes. J.
Phys. Chem. C.

[44] Liu R., Ma L., Huang S., Mei J., Xu J., Yuan G. (2017). A flexible
polyaniline/graphene/bacterial cellulose supercapacitor electrode. New J. Chem..

[45] Zhang X., Lin Z., Chen B., Zhang W., Sharma S., Gu W., Deng Y. (2014). Solid-state flexible polyaniline/silver cellulose nanofibrils aerogel
supercapacitors. J. Power Sources.

[46] Yang C., Li D. (2015). Flexible and foldable
supercapacitor electrodes from the porous 3D
network of cellulose nanofibers, carbon nanotubes and polyaniline. Mater. Lett..

[47] Ge D., Yang L., Fan L., Zhang C., Xiao X., Gogotsi Y., Yang S. (2015). Foldable supercapacitors
from triple
networks of macroporous cellulose fibers, single-walled carbon nanotubes
and polyaniline nanoribbons. Nano Energy.

[48] Yuan T., Zhang Z., Liu Q., Liu X.-T., Miao Y.-N., Yao C.-l. (2023). MXene (Ti3C2Tx)/cellulose
nanofiber/polyaniline film
as a highly conductive and flexible electrode material for supercapacitors. Carbohydr. Polym..

[49] Hu Y., Zhan Y., Xu M., Niu F., Chen Y., Yang Q., Xiong C., Shi Z. (2022). One-step preparation
of flexible nanocellulose-based composite hydrogel supercapacitors
with high specific capacitance. Compos. Sci.
Technol..

[50] Li Y., Xia Z., Gong Q., Liu X., Yang Y., Chen C., Qian C. (2020). Green Synthesis of
Free Standing Cellulose/Graphene Oxide/Polyaniline
Aerogel Electrode for High-Performance Flexible All-Solid-State Supercapacitors. Nanomaterials.

[51] Liu S., Chen Y., Dorsel P.-K. P., Wu C. (2023). Facile preparation
of nanocellulose/multi-walled carbon nanotube/polyaniline composite
aerogel electrodes with high area-specific capacitance for supercapacitors. Int. J. Biol. Macromol..

[52] Xu H., Cui L., Lei Z., Xu M., Jin X. (2022). MXene/carboxymethylcellulose-polyaniline
(Ti3C2Tx/CMC-PANI) film as flexible electrode for high-performance
asymmetric supercapacitors. Electrochim. Acta.

[53] Hsu H. H., Khosrozadeh A., Li B., Luo G., Xing M., Zhong W. (2019). An Eco-Friendly, Nanocellulose/RGO/in
Situ Formed Polyaniline for
Flexible and Free-Standing Supercapacitors. ACS Sustainable Chem. Eng..

[54] Zhang Y., Qi K., Pi X., Tang Q., Xue Q., Chen W., Gu D., Xu J., Zhang S., Wang Y. (2025). Bacterial
cellulose modified polyaniline/Ti3C2Tx MXene fiber to enhance robust
and electrochemical activity for wire-shaped supercapacitors. J. Alloys Compd..

[55] Gharsallah F., Ben Ayed E., Ghorbel N., Putaux J.-L., Boufi S. (2024). Aminated nanocellulose-mediated
synthesis of polyaniline/nanocellulose hybrid materials for sustainable
conductive and supercapacitors nanopapers. Materials
Today Sustainability.

